# An *FGA* Frameshift Variant Associated with Afibrinogenemia in Dachshunds

**DOI:** 10.3390/genes12071065

**Published:** 2021-07-13

**Authors:** Reinhard Mischke, Julia Metzger, Ottmar Distl

**Affiliations:** 1 Clinic for Small Animals, University of Veterinary Medicine Hannover, 30559 Hannover, Germany; reinhard.mischke@tiho-hannover.de; 2 Institute for Animal Breeding and Genetics, University of Veterinary Medicine Hannover, 30559 Hannover, Germany; julia.metzger@tiho-hannover.de

**Keywords:** dog, Dachshund, afibrinogenemia, *fibrinogen alpha chain* gene, association, mutation

## Abstract

Congenital fibrinogen disorders are very rare in dogs. Cases of afibrinogenemia have been reported in Bernese Mountain, Bichon Frise, Cocker Spaniel, Collie, Lhasa Apso, Viszla, and St. Bernard dogs. In the present study, we examined four miniature wire-haired Dachshunds with afibrinogenemia and ascertained their pedigree. Homozygosity mapping and a genome-wide association study identified a candidate genomic region at 50,188,932–64,187,680 bp on CFA15 harboring *FGB* (*fibrinogen beta chain*), *FGA* (*fibrinogen alpha chain*), and *FGG* (*fibrinogen gamma-B chain*). Sanger sequencing of all three *fibrinogen* genes in two cases and validation of the *FGA*-associated mutation (*FGA*:g.6296delT, NC_006597.3:g.52240694delA, rs1152388481) in pedigree members showed a perfect co-segregation with afibrinogenemia-affected phenotypes, obligate carriers, and healthy animals. In addition, the rs1152388481 variant was validated in 393 Dachshunds and samples from 33 other dog breeds. The rs1152388481 variant is predicted to modify the protein sequence of both *FGA* transcripts (FGA201:p.Ile486Met and FGA-202:p.Ile555Met) leading to proteins truncated by 306 amino acids. The present data provide evidence for a novel *FGA* truncating frameshift mutation that is very likely to explain the cases of severe bleeding due to afibrinogenemia in a Dachshund family. This mutation has already been spread in Dachshunds through carriers before cases were ascertained. Genetic testing allows selective breeding to prevent afibrinogenemia-affected puppies in the future.

## 1. Introduction

Congenital fibrinogen disorders represent rare bleeding disorders in humans [1,2,3,4,5,6,7]. They are characterized by deficiency (hypofibrinogenemia, OMIM 202400) or absence of fibrinogen (afibrinogenemia, OMIM 202400) or normal or decreased levels of a dysfunctional fibrinogen (dysfibrinogenemia or hypodysfibrinogenemia, OMIM 616004). In humans, these three different fibrinogen abnormalities are caused by mutations in one of the three fibrinogen genes, *fibrinogen alpha chain (FGA)*, *fibrinogen beta chain (FGB)*, or *fibrinogen gamma chain (FGG)* [2,8]. Hypofibrinogenemia, dysfibrinogenemia, or hypodysfibrinogenemia are autosomal dominant traits with heterozygous or compound heterozygous patients, whereas afibrinogenemia is an autosomal recessive disorder [2]. Each of these three genes encodes a subunit of the fibrinogen protein. Fibrinogen is a glycoprotein with a central role in the final step of the coagulation cascade in hemostasis [9,10]. Mutations not allowing that the fibrinogen protein is assembled result in severe bleeding episodes. Patients with hypofibrinogenaemia, hypodysfibrinogenaemia, or dysfibrinogenaemia are often asymptomatic or show varying symptoms related to fibrinogen levels or reduced activity, whereas afibrinogenemia causes severe bleeding episodes [3]. The *FGA* gene is most often involved in afibrinogenemia and loss of function variants were most frequent [2,11]. Afibrinogenemia manifests often in neonates with umbilical cord bleeding, but onset may also be later in life with mild bleeding abnormalities. Epistaxis, bleeding in the joints, gingival bleeding, menorrhagia, and spontenous abortions are the most common symptoms in patients [12]. 

In dogs, case reports on dysfibrinogenaemia in a Collie and Borzoi [13,14] as well as on hypofibrinogenaemia in the Saint Bernard dog [15] and German Pointer [16] were published. Single cases of afibrinogenemia in the Bernese mountain dog [15], Bichon frise [17], and Chihuahua [18] were reported. In a 1.5-year-old Bichon Frise, a life-threatening hemorrhagic condition occurred due to an antibody inhibitor to fibrinogen secondary to blood transfusion after a surgery [17]. In a 3-year-old female Chihuahua with recurrent bleeding episodes, but, unlike in the Bichon Frise, fibrinogen antibodies were unlikely [18]. Single cases of either hypo- or afibrinogenemia were observed in Vizsla, Collie, Lhasa Apso, Cocker spaniel, and mixed-breed dogs [19,20,21,22,23].

In this study, we present a family of miniature wire-haired Dachshunds segregating for congenital afibrinogenemia. We employed homozygosity mapping, a genome-wide association study (GWAS), and sequencing of fibrinogen genes in order to identify a mutation responsible for afibrinogenemia. Validation in a large sample of all Dachshund breeds and other dogs from many different breeds supported the causal role of the afibrinogenemia-associated mutation. 

## 2. Materials and Methods

### 2.1. Ethics Statement

All animal work has been conducted according to the national and international guidelines for animal welfare. The Lower Saxony state veterinary office at the Lower Saxonian State Office for Consumer Protection and Food Safety (Niedersächsisches Landesamt für Verbraucherschutz und Lebensmittelsicherheit, LAVES), Oldenburg, Germany, was the responsible Institutional Animal Care and Use Committee (IACUC) for this specific study. The EDTA-blood sampling of the Dachshunds for the present study was approved by the IACUC of Lower Saxony, the state veterinary office LAVES, Oldenburg, Germany (registration number 33.9-42502-05-14A465). EDTA-blood samples of control breeds were derived from the bio-bank for diagnostic purposes and were taken from accredited veterinarians. We obtained written informed approval from the dog owners and breeders to use samples and data for current research, publication, and further investigations. 

### 2.2. Animals

Three seven-week-old puppies of miniature wire-haired Dachshunds were presented for a veterinary examination at the Small Animal Clinic of the University of Veterinary Medicine Hannover (cases 1 to 3). These three puppies were littermates and diagnosed with afibrinogenemia. From a male and female (case 1–2), EDTA-blood samples were taken at an age of approximately seven months. An affected female died shortly after hospitalization. We were able to collect EDTA-blood samples from 20 pedigree members of the three cases. A further female 1-year-old dog with excessive bleedings was presented about two years later after the first cases were seen but at another veterinarian clinic. Coagulation diagnostics in our hemostasis laboratory revealed the presence of afibrinogenemia (case 4), and EDTA blood was stored for genotyping. Validation was performed in 393 EDTA-blood samples of adult dogs from all nine Dachshund breeds. These 393 samples and a further 72 EDTA-blood samples were available from our biobank for the present analysis. In all controls, owners assessed and reported the health status of their dogs when shipping a sample to our institute. 

### 2.3. Coagulation Assays

Coagulation assays were measured from citrated plasma (1 part 0.11 mol/L (3.8%) sodium citrate, 9 parts blood) with an automated coagulation analyzer (Amax Destiny Plus, Trinity Biotech, Lemgo, Germany) based on a spheric coagulometric method. Prothrombin time (PT), the screening test of the extrinsic and common pathway, was measured according to the test manual provided by the manufacturer (25 µL of PPP were prewarmed for 60 s, before addition of 50 µL of activating reagent (Thromborel S, Siemens Healthcare Diagnostics Products, Marburg, Germany). In addition, we used a modified method optimized for canine blood. Herein, 25 µL of PPP diluted 1:20 with imidazole buffer (Siemens Healthcare Diagnostics Products, Erlangen, Germany) and 25 µL of human fibrinogen solution (2 g/L human fibrinogen, plasminogen depleted, Enzyme Research Laboratories, Haemochrom Diagnostica, Essen, Germany) were incubated for 120 s before 25 µL of activating reagent (Thromborel S (Enzyme Research Laboratories, Haemochrom Diagnostica)) was added and then time until clot formation was measured. This modified test increases sensitivity of the assay to detect deficiencies of coagulation factors II, V, VII, and X in canine plasma compared to the PT standard test. The outcome of this test is not influenced by reduced fibrinogen concentrations due to the addition of external fibrinogen creating an adequate fibrin clot as a measurement signal.

Measurement of the activated partial thromboplastin time (aPTT), the screening test of the intrinsic and common pathway, was performed with the reagent C.K. Prest (Diagnostica Stago S.A.S., Asnières sur Seine, France) according to the test manual: 25 µL of the activating reagent was added to 25 µL PPP and the mixture was incubated for 180 s, before the coagulation process was initiated by adding 25 µL of 25 mmol/L CaCl_2_ solution (Diagnostica Stago), and coagulation time was measured.

The thrombin time assay (TT) was performed with a commercial reagent containing bovine thrombin (Test thrombin reagent, Siemens Healthcare Diagnostics Products) at 1.5 and 3 U/mL thrombin concentrations resulting in final thrombin concentrations of 1 U/mL and 2 U/mL. Results of this assay depend on the fibrinogen concentration and possible interfering influences on the thrombin-fibrinogen-interaction and the fibrin polymerization.

The fibrinogen concentration was measured coagulometrically according to the Clauss method using a commercially available reagent (Fibri-Prest Automate, Diagnostica Stago) against a standard curve prepared using canine plasma with defined fibrinogen concentrations.

Platelet counts were measured using the hematology system Advia 120 (Siemens Healthineers, München, Germany).

### 2.4. Genotyping and Statistical Analysis

We extracted genomic DNA from the EDTA-blood samples through a standard ethanol fractionation with concentrated sodium chloride (6 M NaCl) and sodium dodecyl sulphate (10% SDS). The concentration of DNA was adjusted to 50 ng/μL per sample. Genotyping was done for two afibrinogenemia-affected (cases 1–2) and 10 unrelated Dachshunds on the canine Illumina high density beadchip containing 173,662 SNPs. Data quality control was done using PLINK v.1.9 (http://www.cog-genomics.org/plink/1.9/, accessed on 27 April 2021) [24]. Data were controlled for Hardy–Weinberg equilibrium (*p*-value < 0.000001), minor allele frequencies (MAF) of >0.05, and a genotyping rate per SNP of >0.95 resulting in 109,257 SNPs for GWAS. Runs of homozygosity were performed on autosomal SNPs and without a MAF-restriction using 165,129 SNPs. The mean genotyping rate was 99 percent.

We employed a sliding window approach for searching ROHs with PLINK v.1.9 (www.cog-genomics.org/plink/1.9/, accessed on 27 April 2021)) [24]. ROHs were called if a homozygous stretch contained 10 or more SNPs and extended over 120 kb. We did not allow for heterozygous SNPs and only for two missing SNPs per homozygous region. ROHs overlapping in the two cases were pooled to consensus ROHs. The number of generations in the past (t) was estimated as t = 1/(2c) with c = size of ROH in Morgan units assuming 1 Mb = 0.01 M [25]. The GWAS was performed using PLINK v1.9 (www.cog-genomics.org/plink/1.9/, accessed on 27 April 2021)) [24].

### 2.5. Sequencing 

Sanger sequencing of genomic DNA of exons and exon/intron boundaries in the three candidate genes *FGA*, *FGB*, and *FGG* was performed in two afibrinogenemia-affected Dachshunds (case 1 and 2) and two unaffected control Dachshunds. In addition, for the confirmation of predicted gene models, cDNA derived from a hair sample stored in RNALater reagent (Qiagen, MA, USA) of one unaffected control dog was sequenced. RNA was isolated and transcribed into cDNA using RNeasy Mini Kit (Qiagen) and Maxima First Strand cDNA Synthesis Kit (Fermentas, Thermo Fisher Scientific, Waltham, MA, USA). We designed primer pairs using Primer3 (http://frodo.wi.mit.edu/primer3/, accessed on 27 April 2021)) for design and UCSC for primer blast (http://genome.ucsc.edu/, accessed on 27 April 2021)). The PCR mastermix was prepared according to standard protocols [19] (Table S3). Reactions were run on a thermocycler TProfessional 96 (Biometra, Göttingen, Germany) for 95 °C for 4 min, 38 cycles of 94 °C for 30 s, primer specific annealing temperature for 30 s, and 72 °C for 45 s. PCR-products were cleaned up using Exonuclease I and FastAP Thermosensitive Alkaline Phosphatase (Thermo Scientific, Darmstadt, Germany) according to the manufacturers’ protocol (http://www.thermoscientificbio.com/dna-and-rna-modifying-enzymes/exonuclease-i, accessed on 27 April 2021)) and sequenced by the service provider GATC Biotech AG (Konstanz, Germany).

### 2.6. Validation and Effect Prediction

The candidate variant *FGA*:g.6296delT (NC_006597.3:g.52240694delA, rs1152388481) was validated using a restriction fragment length polymorphism according to a standard PCR- and digestion-protocol (Table S4). The digestion enzyme MluCI and CutSmart buffer were derived from NEB (NEB BioLabs, Ipswich, MA, USA). Estimations of the functional impacts of all detected variants were obtained by the Variant Effect Predictor [26] for SIFT [27] predictions and PolyPhen-2 [28]. To identify variants which were potentially found in other studies, dbSNP entries were searched for variant positions detected in sequencing analysis [29]. Protein sequence comparisons were performed using Clustal Omega [30].

## 3. Results

### 3.1. Phenotype

**Figure 1:**
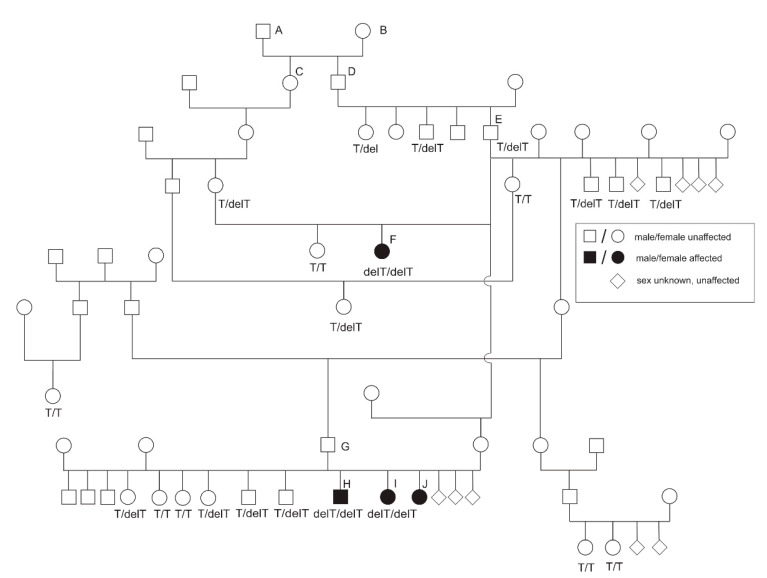
Pedigree of the four afibrinogenemia-affected Dachshunds with their ancestors in common. Sire E has multiple dams as mating partners. Only for the three litters of sire G with three different dams are all siblings given.

Three seven-week-old miniature wire-haired Dachshunds were presented at the Small Animal Clinic of the University of Veterinary Medicine with excessive bleedings after fitting with a chip two days before. Two puppies were female and one male. One female died a few days later due to severe bleedings. The second female puppy survived up to an age of one year. Owners reported recurrent episodes with severe bleedings in the skin and gums. The third affected male puppy is under intensive veterinary care and still alive at the age of seven years despite intermittent severe bleeding episodes. The three dogs (cases 1–3, littermates H, I, J) with severe bleeding episodes were purebred miniature wire-haired Dachshunds according to FCI (Fédération Cynologique Internationale) standards (Figure 1). None of the other three littermates or parents showed signs of increased bleeding tendency. Case 4 (animal F) died from a hemoabdomen after a traumatic splenic rupture. Its pedigree data showed a relationship with cases 1–3 through its sire (animal E). Case 4 was inbred on ancestors A and B and cases 1–3 on ancestor E (Figure 1). All four affected animals shared the most recent common ancestry with animals A and B in the pedigree. 

Other littermates, parents, and further related dogs did not show any signs of increased bleeding tendency. 

In the affected dogs, PT (standard test), aPTT, and TT exceeded the upper limits of detection (>200 s). PT measured with the optimized assay revealed increased or normal activities of factors II, V, VII, and X, respectively (case 1: 149%; case 2: 104%; case 3: 126%; 100% = average of normal adult dogs) and a moderate reduction in case 4 (44%, reference range in adult dogs: 75–130%). Fibrinogen concentration according to the coagulometric Clauss method was below the lowest detection limit in all four cases (<0.2 g/L; reference 1.0–3.0 g/L). Platelet count was normal in all four affected animals suffering from bleeding complications.

A diagnosis of afibrinogenemia was suspected based on PT, aPTT, and TT results and confirmed by the Clauss method. 

### 3.2. Homozygosity Mapping and Genome-Wide Association

Genotyping on the canine Illumina high density beadchip with 173,662 single nucleotide polymorphisms (SNPs) was performed for two puppies surviving the first year of life (cases 1–2) and 10 unrelated Dachshunds as controls. Homozygosity mapping revealed 63 consensus regions on the reference genome CanFam 2.0 for the two affected Dachshunds (Supplementary Table S1). On CFA15, the consensus region at 53,204,069–67,208,667 bp (14,004.6 kb) on CanFam 2.0 (50,188,932–64,187,680 on CanFam 3.1) contained the genes *FGB* (*fibrinogen beta chain*) at 55,242,068–55,251,060 bp (52,220,662–52,229,692 bp, CanFam 3.1), *FGA* (*fibrinogen alpha chain*) at 55,261,815–55,268,417 bp (52,234,319–52,246,920 bp, CanFam 3.1) and *FGG* (*fibrinogen gamma-B chain*) at 55,283,023–55,291,196 bp (52,261,220–52,270,169 bp, CanFam 3.1). Further potential candidate genes were not identified within the homozygous consensus regions. Other potential candidate genes on CFA2 (*FCN3* (*ficolin 3*) at 72,900,744–72,903,639 bp, CanFam 3.1), CFA9 (*FCN2* (*ficolin 2*) at 50,868,942–50,876,001 bp, *FIBCD1* (*fibrinogen C domain-containing 1*) at 53,073,859–53,106,266 bp, CanFam 3.1), CFA16 (*FGL1* (*fibrinogen-like 1*) at 41,161,314–41,191,282 bp, CanFam 3.1), and CFA18 (*FGL2* (*fibrinogen-like 2*) at 17,328,794–17,335,429 bp, CanFam 3.1) were outside the homozygous consensus regions identified. 

A GWAS using PLINK, version 1.9 (http://pngu.mgh.harvard.edu/purcell/plink/, accessed on 27 April 2021)) revealed a significant association at 33,377,925–59,552,763 bp on CFA15 (30,364,874–56,533,077 bp, CanFam 3.1) comprising 16 SNPs with raw *p*-values of 9.634 × 10^−7^ and spanning the homozygosity region containing *FGA*, *FGB* and *FGG* (Supplementary Table S2). Both affected animals had the same homozygous genotype in the associated SNPs, and controls were homozygous for the alternate allele for each SNP. Other SNPs within the homozygous consensus regions did not reach FDR-corrected *p*-values < 0.05.

### 3.3. Candidate Gene Sequencing

**Figure 2:**
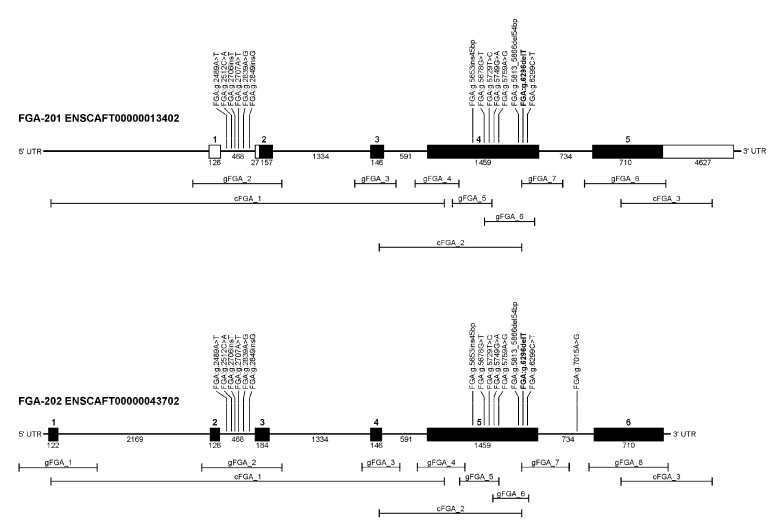
FGA gene models. Gene structure of two *FGA* transcripts including exons, introns, and detected variants are shown. PCR-products were designed for sequencing of exon–intron boundaries in genomic DNA and coding regions in complementary DNA. Amplicon sizes of genomic DNA and cDNA are depicted below the respective gene models.

Sanger sequencing of canine complementary DNA (cDNA) of a control dog confirmed the gene model with two transcripts in *FGB* (ENSCAFT00000035915/ENSCAFT00000013386), two transcripts in *FGG* (ENSCAFT00000013420/XM_532698.5), and three transcripts in *FGA* (ENSCAFT00000043702/ENSCAFT00000013402/unknown), of which one transcript was not in the Ensembl or NCBI database (Figure 2; Supplementary Figures S1 and S2). 

**Figure 3:**
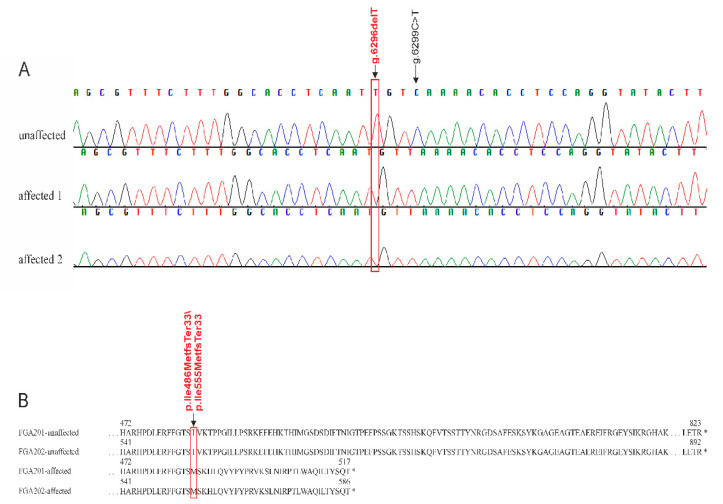
Frameshift mutation in afibrinogenemia-affected Dachshunds. Nucleotide key for the respective individual is given above the chromatogram. Chromatograms from sequencing analysis show the frameshift variant *FGA*:g.6296delT (rs1152388481) in both affected dogs (affected 1–2, identical to case 1–2; (**A**)). Comparisons of the predicted protein sequence of *FGA* transcripts (*FGA201* and *FGA202*) in affected and unaffected dogs display a significant shortening of the amino acid sequence due to the frameshift mutation (**B**).

Sequencing exons with their intron boundaries of *FGA*, *FGB*, and *FGG* genes in the two affected dogs (cases 1–2) and two unaffected controls revealed 11 SNPs and five INDELs in *FGA*, one SNP in *FGB*, and five SNPs and one INDEL in *FGG* (Table 1). Among these polymorphisms, two intronic variants (*FGA*:g.2512C>A, *FGA*:g.2839A>G), two synonymous variants (*FGA*:g.5678G>T, *FGA*:g.6299C>T), and one frameshift variant (*FGA*:g.6296delT) detected in *FGA* were exclusively homozygous mutant in both of these affected dogs. Validation of these five SNPs in the miniature wire-haired Dachshund family with the four cases showed only *FGA*:g.6296delT (rs1152388481, NC_006597.3:g.52240694delA, ENSCAFT00000013402.3:c.1602del, ENSCAFT00000043702.3:c.1665del) in perfect co-segregation with the afibrinogenemia phenotype (Figure 1). Chromatograms from sequencing analysis show the *FGA* frameshift variant rs1152388481 for cases 1 and 2 in comparison to a control dog (Figure 3A). Predicted protein sequences of *FGA* transcripts *FGA201* and *FGA202* in an affected dog display a shortening of the amino acid sequence due to the frameshift mutation (Figure 3B). The sire of the three affected puppies (cases 1–3) had three litters with three different dams and, in each of the three litters, progeny with the mutant allele were found. Parents of case 4 were heterozygous mutants. For the sire of case 4 (animal E in Figure 1), we found two heterozygous fullsiblings and three heterozygous mutant progeny in two different litters. In this pedigree, heterozygous mutant genotypes were observed in nine litters. All animals with the mutant *FGA* rs1152388481 variant shared a common ancestry with the parent couple (animal A and B). An X-linked inheritance is very unlikely as three female progeny of unaffected dams and sires showed the afibrinogenemia phenotype. 

### 3.4. Genotyping of the Candidate FGA Variant

Further validation of the frameshift variant *FGA*:g.6296delT (rs1152388481) in 393 healthy Dachshunds of different breeds revealed a perfect co-segregation of the phenotypes for afibrinogenemia (Table 2). None of the unaffected Dachshunds showed the homozygous mutant genotype of the rs1152388481 variant. In total, three genetic carriers were detected in wire-haired Dachshunds and wire-haired Miniature Dachshunds. The three Dachshunds with a heterozygous mutant rs1152388481 genotype were related to the afibrinogenemia-affected dogs investigated in this study through their maternal grandsire (animal D in Figure 1, two animals) and its paternal grandsire (animal E in Figure 1, one animal). Additional genotyping of 72 samples of 33 different dog breeds revealed that this variant was private in Dachshunds and not present in any other examined dog breed (Table S5). 

## 4. Discussion

Congenital fibrinogen disorders are extremely rare disorders in dogs and only very few detailed descriptions of cases were reported [15,16,18]. The extremely prolonged (non-measurable) clotting times of all three group tests (PT, aPTT, TT) are indicative for the nearly complete lack of fibrinogen [23], and results of TT excluded anticoagulant rodenticide toxicity. The modified PT test indicated normal or increased activities of factors II, V, VII, and X, except in case 4. The reduced coagulation factor activity in that dog of 44% may reflect a dilution coagulopathy, most likely due to the hemoabdomen. The normal or increased activities of factors II, V, VII, and X excluded liver failure wherefore, in addition, no other indications were found. Severe hyperfibrinolysis either primary or more commonly secondary in association with disseminated intravascular coagulation (DIC) appeared unlikely. Underlying diseases possibly triggering DIC and/or hyperfibrinolysis were not likely as platelet counts were normal. Measurements of fibrinogen degradation products and D dimers may have given additional information. A further rare cause of afibrinogenemia are IgG antibodies against subunits of the fibrinogen molecule, which were detected in a Bichon Frise dog secondary to blood transfusion [17]. Although not assessed in our patients, such an autoimmune reaction was unlikely, as treatments with blood products were not done. 

We showed a 1-bp deletion causing a frameshift mutation of the coding sequence of the *FGA* gene with a predicted truncation of the FGA protein. Afibrinogenemic puppies were homozygous mutant for the *FGA* rs1152388481 variant supporting that this mutation is responsible for afibrinogenemia. Validation of the *FGA* rs1152388481 variant in the pedigree of the cases, in dogs of all nine Dachshund breeds and a large panel of dogs from other breeds, gave confidence that this *FGA*-associated mutation is responsible for afibrinogenemia in these Dachshunds. It seems likely from our data that the *FGA* rs1152388481 variant was segregating for at least four generations. A heterozygous male breeding dog (animal E) sired an affected animal (case 4) and heterozygous progeny in two different litters, and this breeding dog was identified as a common ancestor for cases 1–3. All dogs of the ascertained pedigree had full pedigree records fulfilling FCI standards. Therefore, we were able to screen pedigree data for common ancestors on all possible paths. These data allowed us to trace back all animals carrying the afibrinogenemia variant *FGA* rs1152388481 to the most recent common parent couple (animal A and B) and possibly to the origin of this mutant variant in this pedigree. The size of the homozygosity region surrounding the fibrinogen genes is 14 Mb. An estimate of when the mutation arose can be derived from the size of the homozygosity region surrounding the fibrinogen genes with t = 1/(2c), with t = number of generations and c = 100 Mb~1 Morgan [25]. With a size of 14 Mb of the homozygosity region, we obtained an estimate of four generations. This may indicate that the *FGA* rs1152388481 variant was due to a novel mutation in one of the sires or dams of the most recent common parent couple. This couple had no ancestors in common for at least three generations. The low number of cases observed in Dachshunds is in agreement with this estimate. An autosomal recessive mode of inheritance is supported by the segregation pattern of phenotypes and genotypes observed in the pedigree. Severe clinical cases with the absence of fibrinogen and a recessive inheritance for afibrinogenemia are in agreement with observations in human medicine [1,2,3,4,5]. In addition, a loss of functional variants are common in human afibrinogenemic patients [2,3,11]. 

The mechanisms of nonsense mutations of the afibrinogenemia causing variants were studied in cell transfection experiments [5]. These studies did not give evidence for nonsense mediated decay of mutant mRNA. Expression studies with transfected cells performed for a *FGG* nonsense mutation causing afibrinogenemia in a homozygous mutant state of patients (p.Arg134X) showed no difference in mRNA levels between wildtype and mutant cells [5]. For *FGB* nonsense mutations (p.Trp467X and p.Trp470X), synthesis and intracellular assembly of the protein chain were observed in experiments with transfected cells, but secretion was impaired [5]. Thus, impaired secretion of fibrinogen was suggested to cause afibrinogenemia in these reported homozygous mutant *FGB- and FGG-*cases. For the present cases with the *FGA* rs1152388481 variant, similar experiments were not performed. 

## 5. Conclusions

In conclusion, we identified a novel and likely recently arisen mutation for afibrinogenemia in a Dachshund pedigree. This is the first mutation identified for a congenital fibrinogen disorder in dogs and even in a domestic animal species. The mechanism of the *FGA* rs1152388481 variant resulting in afibrinogenemic puppies remained unknown. Knowledge of the *FGA*-associated mutation in Dachshunds allows genetic testing of dogs of Dachshund breeds to prevent this lethal disease. This genetic test may also serve as an additional tool for clinical diagnosis of afibrinogenemia in Dachshunds. Breeders are now able to eliminate this defective allele in their Dachshund breeding lines.

## Figures and Tables

**Table 1:** Variants detected in sequencing analysis of the candidate genes *FGA*, *FGB*, and *FGG*. The predicted effect on gene sequence, genotypes of afibrinogenemic dogs and two controls, and SIFT predictions are shown.

Polymorphism ID	dbSNP-ID	Predicted Effect	Base Change	Genotype Affected Dog 1	Genotype Affected Dog 2	Genotype Reference Dog 1	Genotype Reference Dog 2	SIFT Prediction
*FGA:g.2489A>T*	rs22378576	intron variant	-	T/T	T/T	T/T	A/A	-
*FGA:g.2512C>A*	rs22378571	intron variant	-	A/A	A/A	C/A	C/A	-
*FGA:g.2706insT*	-	intron variant	-	ins/ins	ins/ins	ins/ins	del/ins	-
*FGA:g.2707A>T*	-	intron variant	-	T/T	T/T	T/T	T/T	-
*FGA:g.2839A>G*	rs22378570	intron variant	-	G/G	G/G	G/A	A/A	-
*FGA:g.2849insG*	-	intron variant	-	ins/ins	ins/ins	ins/ins	ins/ins	-
*FGA:g.5653insTAGTGCTGGTACTTGGAGCACCAGGCCTGGCAGCACTGGGCCTGG*	-	in-frame insertion	-	ins/ins	ins/ins	ins/ins	ins/ins	-
*FGA:g.5678G>T*	rs850697053	synonymous	-	T/T	T/T	G/T	G/T	-
*FGA:g.5729T>C*	rs851893391	synonymous	-	C/C	C/C	C/C	C/C	-
*FGA:g.5749G>A*	rs852841801	missense	S/N	A/A	A/A	G/G	A/A	deleterious low confidence (0.02)
*FGA:g.5759A>G*	rs851142303	synonymous	-	G/G	G/G	A/A	G/G	-
*FGA:g.5812T>C*	rs851802762	missense	L/P	C/C	C/C	C/C	C/C	tolerated low confidence (0.63/0.65)
*FGA:g.5813_5866delGGGCAGTACTGGCACTTGGAGCTCCGGGAGCACCGGGCCTGGCAACACTGGACC*	-	in-frame deletion	-	del/del	del/del	del/del	del/del	-
*FGA:g.6296delT*	rs1152388481	deletion -frameshift	-	del/del	del/del	T/T	T/T	-
*FGA:g.6299C>T*	rs852430931	synonymous	-	T/T	T/T	C/C	C/C	-
*FGA:g.7015A>G*	-	intron variant	-	G/G	G/G	G/G	G/G	-
*FGG:g.169T>C*	rs8695728	intron variant	-	C/C	C/C	C/C	T/C	-
*FGG:g.383insA*	-	intron variant	-	ins/ins	ins/ins	ins/ins	ins/ins	-
*FGG:g.391A>C*	-	intron variant	-	A/A	A/A	A/A	A/C	-
*FGG:g.469T>C*	rs8695727	intron variant	-	C/C	C/C	C/C	C/C	-
*FGG:g.578T>C*	rs8695726	synonymous	-	C/C	C/C	C/C	T/C	-
*FGG:g.754T>C*	rs22423864	intron variant	-	C/C	C/C	C/C	C/C	-
*FGB:g.7827+93C>T*	rs22380419	3′UTR variant	-	T/T	T/T	T/T	T/T	-

**Table 2:** Genotypic distribution of *FGA* rs1152388481 variant in controls including 393 healthy Dachshunds (N = number of animals, T = wildtype allele, delT = deletion of T).

Breed	N	T/T	T/delT	delT/delT
Standard Dachshund—smooth-haired	22	22	0	0
Standard Dachshund—wire-haired	211	209	1	0
Standard Dachshund—long-haired	17	17	0	0
Miniature Dachshund—smooth-haired	11	11	0	0
Miniature Dachshund—wire-haired	75	67	2	0
Miniature Dachshund—long-haired	18	18	0	0
Rabbit Dachshund—smooth-haired	8	8	0	0
Rabbit Dachshund—wire-haired	25	25	0	0
Rabbit Dachshund—long-haired	4	4	0	0

## Data Availability

The genotyping data on the canine Illumina high density beadchip used for the current study are available from the corresponding author upon a reasonable request.

## Supplementary Materials

The following are available online at https://www.mdpi.com/article/10.3390/genes12071065/s1, Figure S1: *FGB* gene model. Gene structure of two *FGB* transcripts including exons, introns, and detected variants are shown. PCR-products were designed for sequencing of exon–intron boundaries in genomic DNA and coding regions in complementary DNA. Figure S2: *FGG* gene model. Gene structure of the two detected *FGG* transcripts from NCBI and Ensembl database including exons, introns, and detected variants are shown. PCR-products were designed for sequencing of exon–intron boundaries in genomic DNA and coding regions in complementary DNA. Table S1: Homozygous consensus regions identified in the two affected Dachshunds using genotypes of the canine Illumina high density beadchip with 173,662 single nucleotide polymorphisms (SNPs). Chromosomal positions of the homozygosity regions with their delimiting SNPs at the proximal and distal ends, size of the homozygosity regions in kb, number of SNPs, and potential candidate genes within the homozygosity regions are shown. Table S2: Afibrinogenemia-associated SNPs within the homozygosity regions with their raw *p*-values, Bonferroni-corrected *p*-values (*p*-value-Bon), and *p*-values corrected for false discovery rate (*p*-value-FDR) and candidate genes contained within the homozygosity region at 55.242–55.291 Mb on dog chromosome (CFA) 15. Table S3: Primer sequences for genomic and complementary DNA sequencing of *FGA*, *FGB*, and *FGG*. Targeted genes, primers, product sizes in base pairs (bp), and annealing temperatures (AT) are shown. Table S4: Validation of the candidate variant *FGA*:g.6296delT (rs1152388481) using a restriction fragment length polymorphism (RFLP). Primer pairs, annealing temperature (AT), restriction enzyme, and incubation temperature (IT) for validation of the frameshift variant *FGA*:g.6296delT (rs1152388481) are shown. Table S5: Genotypic distribution of the *FGA*:g.6296delT (rs1152388481) variant in controls including 72 healthy dogs of breeds other than Dachshunds.
