# Supplementary material for: An FGA Frameshift Variant Associated with Afibrinogenemia in Dachshunds
Source: Genes (Basel). 2021 Jul 13;12(7):1065. doi: 10.3390/genes12071065 (PMC8304930; doi:10.3390/genes12071065)
Supplement: Supplementary file 1 [file genes-12-01065-s001.zip › Table_S1_ROH.pdf]

genes MDPI

Table S1. Homozygous consensus regions identified in the two affected Dachshunds using genotypes of the canine Illumina high density beadchip with 173,662 single nucleotide polymorphisms (SNPs). Chromosomal positions of the homozygosity regions with their delimiting SNPs at the proximal and distal ends, size of the homozygosity regions in kb, number of SNPs and potential candidate genes within the homozygosity regions are shown.

CFA	Start	End	Start	End	Size in kb	Number of SNPs	Candidate genes
	Locus	Locus	Position (bp)	Position (bp)			
1	BICF2P970528	BICF2P313176	3965253	7501259	3536.010	253	
2	BICF2S23624171	BICF2P1442765	28398104	29213561	815.457	60	
2	BICF2P471986	BICF2P1039597	80733586	81960271	1226.680	111	
3	BICF2P288807	BICF2S23716361	65118122	65782820	664.698	60	
3	BICF2S2335282	BICF2G630354100	86322701	87158880	836.179	71	
3	BICF2G630358871	BICF2G630359688	91468680	92023901	555.221	59	
5	BICF2G630183899	BICF2G630185017	37445259	39703097	2257.840	169	
5	BICF2P151703	BICF2G630186354	40692078	41737933	1045.860	84	
6	BICF2G630577427	BICF2P1060954	51808173	53856926	2048.750	173	
7	BICF2P1193290	BICF2P972190	43703042	44387496	684.454	49	
7	BICF2P714978	BICF2G630563620	54834751	64362748	9528.000	750	
7	BICF2G630564734	BICF2S22935480	65286034	66266852	980.818	76	
7	BICF2P486868	BICF2S23319884	81758895	82973110	1214.210	101	
8	BICF2P1251769	BICF2P769851	44734983	45392329	657.346	48	
9	BICF2P1318954	TIGRP2P242449_RS8698184	16590986	20150433	3559.450	257	
9	BICF2P524622	BICF2P20832	30651594	34039241	3387.650	274	
9	BICF2G630834951	BICF2G630835522	34947381	36110703	1163.320	86	
9	BICF2G630472906	BICF2P375985	55722536	56315572	593.036	52	
10	BICF2P401145	BICF2S23248674	68184234	68968385	784.151	73	
10	BICF2P440252	BICF2P1303845	70519661	70555546	35.885	3	
13	BICF2G630603376	BICF2G630603497	5744993	6698034	953.041	74	
13	BICF2P37931	BICF2P565100	16498449	20260365	3761.920	302	
13	BICF2G630611849	BICF2G630614021	21544303	24286245	2741.940	236	
13	BICF2P918987	BICF2P514080	31213047	32676178	1463.130	141	
14	BICF2P50680	BICF2P703887	19404541	20729536	1324.990	103	
14	BICF2P1341234	BICF2P370759	41352763	41435796	83.033	11	
14	BICF2P346885	BICF2S23619817	43281546	45336230	2054.680	164	
15	BICF2G630437135	BICF2G630433012	26619845	32936590	6316.740	487	
15	BICF2G630432944	BICF2G630428314	33064733	50383479	17318.700	1277	
15	TIGRP2P203617_RS9013270	BICF2G630416960	53204069	67208667	14004.600	1123	*FGA, FGB, FGG*
16	BICF2P964713	BICF2G630115080	26921521	37199636	10278.100	779	
16	BICF2S22954267	TIGRP2P216709_RS9075564	44160603	45334803	1174.200	92	
17	BICF2P1438690	BICF2G630213153	23437395	31179935	7742.540	591	
17	BICF2G630211502	BICF2G630209844	34309084	36661407	2352.320	197	
19	BICF2P1194743	BICF2G63042179	19689349	22783900	3094.550	233	
19	TIGRP2P263010_RS9002257	BICF2G630253458	23380530	53110345	29729.800	2389	
20	BICF2P511692	BICF2S23518579	6078437	6807884	729.447	65	
20	BICF2S2297949	BICF2P1091780	32202618	32338543	135.925	9	
20	BICF2P1421842	BICF2P351086	36676605	37357365	680.760	53	
21	BICF2S2343635	BICF2S22945125	9396521	15309519	5913.000	466	
22	BICF2G630324101	BICF2P1252875	25703780	26296220	592.440	49	
23	BICF2S23333750	TIGRP2P300678_RS8947894	16952214	17603862	651.648	56	

23	BICF2G630374109	BICF2P1006996	34827224	36528734	1701.510	126
24	TIGRP2P311568_RS8818767	BICF2P772634	16188989	30498927	14309.900	1161
24	BICF2S23438280	BICF2S23416533	33513575	34097078	583.503	53
25	BICF2P1001256	BICF2G63095922	28921495	30164948	1243.450	98
25	BICF2P1158262	TIGRP2P328076_RS8791149	30483517	31165997	682.480	63
26	BICF2P568282	BICF2G630805684	8169693	13765506	5595.810	422
26	BICF2P1095733	BICF2P1090985	31035792	32032759	996.967	74
26	BICF2S23612664	BICF2S23730355	36082856	37146830	1063.970	91
28	BICF2P507432	BICF2S23613252	31401269	32524848	1123.580	90
28	BICF2S2331225	BICF2P242217	35724033	36707541	983.508	84
28	BICF2G630261607	BICF2P1468079	40262818	40567398	304.580	28
29	BICF2P1433782	BICF2P209589	3039909	10875665	7835.760	562
29	BICF2S23612214	BICF2P1166557	30719444	30928646	209.202	14
30	BICF2S23246075	BICF2P719614	15573683	25294966	9721.280	733
30	BICF2G630405263	BICF2S22959687	25349448	26707357	1357.910	125
32	BICF2G630601412	BICF2P588402	7073405	8287483	1214.080	96
33	BICF2G63076186	TIGRP2P390847_RS8628478	30951092	34409159	3458.070	273
36	BICF2P676634	BICF2P1120783	25968152	27788871	1820.720	153
37	BICF2P1325808	BICF2P1291340	4199639	5315815	1116.180	88
37	BICF2P126631	BICF2P1153945	13957789	19607211	5649.420	413
38	BICF2P203234	BICF2S23048313	16967539	17679244	711.705	62
