# Supplementary material for: An FGA Frameshift Variant Associated with Afibrinogenemia in Dachshunds
Source: Genes (Basel). 2021 Jul 13;12(7):1065. doi: 10.3390/genes12071065 (PMC8304930; doi:10.3390/genes12071065)
Supplement: Supplementary file 1 [file genes-12-01065-s001.zip › Table_S2_asso-SNPs.pdf]

genes MDPI

Table S2. Afibrinogenemia-associated SNPs within the homozygosity regions with their raw P-values, Bonferroni-corrected P-values (P-value-Bon) and P-values corrected for false discovery rate (P-value-FDR) and candidate genes contained within the homozygosity region at 55.242-55.291 Mb on dog chromosome (CFA) 15. For other homozygosity regions, significantly associated SNPs were not detected.

CFA	SNP	Position in bp (CanFam 2.0)	Candidate genes	Raw P-value	P-value-Bon	P-value-FDR
15	BICF2S23711145	33,377,925		9.634e-07	0.1053	0.0009837
15	BICF2G630432361	33,825,951		9.634e-07	0.1053	0.0009837
15	BICF2G630429818	48,050,236		9.634e-07	0.1053	0.0009837
15	BICF2G630429281	48,955,739		9.634e-07	0.1053	0.0009837
15	BICF2P175978	49,084,998		9.634e-07	0.1053	0.0009837
15	BICF2G630429065	49,207,246		9.634e-07	0.1053	0.0009837
15	BICF2G630428910	49,405,394		9.634e-07	0.1053	0.0009837
15	BICF2G630428897	49,419,412		9.634e-07	0.1053	0.0009837
15	BICF2P36775	54,004,399		9.634e-07	0.1053	0.0009837
15	BICF2G630425883	55,913,159	*FGA, FGB, FGG*	9.634e-07	0.1053	0.0009837
15	BICF2P201604	56,127,210		9.634e-07	0.1053	0.0009837
15	BICF2P1347147	57,359,196		9.634e-07	0.1053	0.0009837
15	BICF2G630422858	59,042,979		9.634e-07	0.1053	0.0009837
15	BICF2S2354696	59,062,351		9.634e-07	0.1053	0.0009837
15	BICF2G630422437	59,538,132		9.634e-07	0.1053	0.0009837
15	BICF2G630422419	59,552,763		9.634e-07	0.1053	0.0009837
