# Supplementary material for: An FGA Frameshift Variant Associated with Afibrinogenemia in Dachshunds
Source: Genes (Basel). 2021 Jul 13;12(7):1065. doi: 10.3390/genes12071065 (PMC8304930; doi:10.3390/genes12071065)
Supplement: Supplementary file 1 [file genes-12-01065-s001.zip › Table_S3_Primer.pdf]

genes MDPI

Table S3. Primer sequences for genomic and complementary DNA sequencing of *FGA*, *FGB* and *FGG*. Targeted genes, primers, product sizes in base pairs (bp) and annealing temperatures (AT) are shown.

Gene	Primer name	Forward primer (5'-3')	Reverse primer (5'-3')	Product size (bp)	AT (°C)	genomic or coding region
FGA	gFGA_1	GTACTCGAGCAGGGGTACATC	TTAAAAACACACACCCACACC	848	58	genomic
FGA	gFGA_2	AATGCTGTTCTCTTTCGTGTG	TTGGTTGTTTCTCTGTTGTGC	873	58	genomic
FGA	gFGA_3	AAGCAAATCACTTAGCTGTTCC	CAATGTCCACCTAGAGAGAAGG	801	58	genomic
FGA	gFGA_4	CTCCAAGTCAGAGAAATGAACC	TTCGGAGTCAGAACTTCCAG	877	58	genomic
FGA	gFGA_5	CATCTCATGGAACAGGATCAG	GGTGTTTTGACAATTGAGGTG	853	58	genomic
FGA	gFGA_6	TTGGAGCTCTGGAAGTTCTG	CTTTAGCATGGCCTCTTTTG	704	58	genomic
FGA	gFGA_7	TGATCTAGAGCGTTTCTTTGG	TAAATTGCAAAGGGGATTTTC	848	58	genomic
FGA	gFGA_8	CTTCCTGTCTTCATTTAATCTGG	TTTTCTTCTTCACCTCCCTAAAC	852	58	genomic
FGA	cFGA_1	TTCCTTTCTTTCAGCTGGAGTGC	TCTAAAGCCCTACTGCATGACC	620	59	coding
FGA	cFGA_2	AAATTGAGATCCTGAGGCGC	TGACACCTCTTCAAATGTCCCC	947	59	coding
FGA	cFGA_3	TTTTAACCGGACCTGGCAAG	TCTTCTTCACCTCCCTAAACAAAG	600	59	coding
FGB	gFGB_1	TTTCTTTCTCTCGTTCCTGAAG	ATCCTGTAGGACACAACACTCC	877	58	genomic
FGB	gFGB_2	CTGATGTTTCTTGTCATGATGG	TAAGAGCAGTGGGAGCTATTTG	785	58	genomic
FGB	gFGB_3	CTCAGCAAATCAAAATCACAAC	AATTTCCTCACATTCTGCAAAC	865	58	genomic

Table S3 continued.

Gene	Primer name	Forward primer (5'-3')	Reverse primer (5'-3')	Product size (bp)	AT (°C)	genomic or coding region
FGB	gFGB_4	CAATCCTGGAAAACTTGAGAAG	GGTTAAGTTCCAAATCAAATGG	880	58	genomic
FGB	gFGB_5	TGGTTAATATGTGGCATGTTTG	AGAGCTCAGTATGGTGGCTAAG	801	58	genomic
FGB	gFGB_6	TTTGCAAAGAATAACTCGATAGC	AATGGCAGTGTAGTGGTTTTTC	857	58	genomic
FGB	gFGB_7	GAAGATTCAAAATCTCGCTCTG	GGGGAAAGAGGCTATTAAAATG	816	58	genomic
FGB	gFGB_8	TTCCCATAAACTCTGTCTCACC	AGGTATGGTCTCTGTGTTCCTG	815	58	genomic
FGB	cFGB_1	CAGTAAAGTCCACCAGCAAAGG	CGGCAGTATTCCATCTGAGC	684	59	coding
FGB	cFGB_2	ACTAACCTTCGTGTGCTCCG	CATTGTCTCTGTCGTACGTGC	569	59	coding
FGB	cFGB_3	AAGGGAACAGCTGGCAATGC	TCTGAACAAGGACAGAGAATTCC	661	59	coding
FGG	gFGG_1	TGCTTCACAGGAGAAAGTTTG	TGATCACTTCTTTGGCTTCTG	859	58	genomic
FGG	gFGG_2	GGGTAGTTATTGCCCGACTAC	AAGTGGGGAAAGGTAAAAAGG	485	58	genomic
FGG	gFGG_3	AATTAGTCCCCTGAAAGGAATC	TCACTATGTGACCTTCAGCAAG	754	58	genomic
FGG	gFGG_4	GTTATCCTGCAACAACCCTTAG	TGACCTAGGGAAAGGTAATCTG	742	58	genomic
FGG	gFGG_5	ATTTTTAGATGTCACTGGAATGC	ACTGGGTGGATTTCTAATAGAGG	716	58	genomic
FGG	gFGG_6	CGTACTTTATGGACTTTCAGAGG	TCTTAAAATGAAGTGAGGCTTTG	870	58	genomic
FGG	gFGG_7	ACCAGAACACCATGTTGAAATAC	AGGAAAGTGATGGTTTTTAATGG	767	58	genomic
FGG	cFGG_1	GCACCTAGACACCATGACTTG	TGTCTTGACAATCTTTCCCAGTTG	557	59	coding

Table S3 continued.

Gene	Primer name	Forward primer (5’-3’)	Reverse primer (5’-3’)	Product size (bp)	AT (°C)	genomic or coding region
FGG	cFGG_2	GCAAAGTGTCAGGAACCTTGC	TCCACCAACCAGATCCATCC	610	59	coding
FGG	cFGG_3	CCACAATGGCATGCAGTTCA	AATGTTTATTGAAATGGCCTGTTGA	511	59	coding
FGG	cFGG_4	CCACAATGGCATGCAGTTCA	ACTCCAAGTTCAGTGATTTGAGAA	517	59	coding
