# Supplementary material for: An FGA Frameshift Variant Associated with Afibrinogenemia in Dachshunds
Source: Genes (Basel). 2021 Jul 13;12(7):1065. doi: 10.3390/genes12071065 (PMC8304930; doi:10.3390/genes12071065)
Supplement: Supplementary file 1 [file genes-12-01065-s001.zip › Table_S4_RFLP.pdf]

genes

MDPI

Table S4. Validation of the candidate variant *FGA*:g.6296delT (rs1152388481) using a restriction fragment length polymorphism (RFLP). Primers, annealing temperature (AT), restriction enzyme and incubation temperature (IT) for validation of the frameshift variant *FGA*:g.6296delT are shown.

**Gene**	**Polymorphism**	**Forward primer (5'-3')**	**Reverse primer (5'-3')**	**AT (°C)**	**Restriction enzyme**	**IT (°C)**
FGA	*FGA*:g.6296delT	TTGGAGCTCTGGAAGTTCTG	CTTTAGCATGGCCTCTTTTG	58	MluCI	37
