# Supplementary material for: An FGA Frameshift Variant Associated with Afibrinogenemia in Dachshunds
Source: Genes (Basel). 2021 Jul 13;12(7):1065. doi: 10.3390/genes12071065 (PMC8304930; doi:10.3390/genes12071065)
Supplement: Supplementary file 1 [file genes-12-01065-s001.zip › Table_S5_validation.pdf]

genes

MDPI

Table S5. Genotypic distribution of the *FGA*:g.6296delT (rs1152388481) variant in controls including 72 healthy dogs of breeds other than Dachshunds.

Breed	N	T/T	T/delT	delT/delT
Afghan Hound	7	7	0	0
Akita	4	4	0	0
American Bulldog	1	1	0	0
American Staffordshire Terrier	1	1	0	0
Australian Shepherd	2	2	0	0
Beagle	2	2	0	0
Belgian Shepherd Dog	1	1	0	0
Bernese Mountain Dog	1	1	0	0
Borzoi	5	5	0	0
Briard	2	2	0	0
English Cocker Spaniel	2	2	0	0
Entlebuch Dog	1	1	0	0
French Bulldog	5	5	0	0
German Shepherd Dog	2	2	0	0
German Wirehaired Pointer	1	1	0	0
Golden Retriever	1	1	0	0
Great Dane	3	3	0	0
Hanoverian Scenthound	2	2	0	0
Hovawart	2	2	0	0
Italian Greyhound	3	3	0	0
Kuvasz	3	3	0	0
Labrador Retriever	1	1	0	0
Newfoundland	3	3	0	0
Polish Lowland Sheepdog	1	1	0	0
Pyrenean Mountain Dog	2	2	0	0
Rhodesian Ridgeback	2	2	0	0
Romagna Water Dog	2	2	0	0
Rough Collie	1	1	0	0
Saluki	1	1	0	0
Samoyed	3	3	0	0
Shiba Inu	2	2	0	0
Tibetan Terrier	1	1	0	0
Weimaraner	2	2	0	0
