# Supplementary figures and images for: An FGA Frameshift Variant Associated with Afibrinogenemia in Dachshunds
Source: Genes (Basel). 2021 Jul 13;12(7):1065. doi: 10.3390/genes12071065 (PMC8304930; doi:10.3390/genes12071065)

**FGB-201 ENSCAFT00000035915**

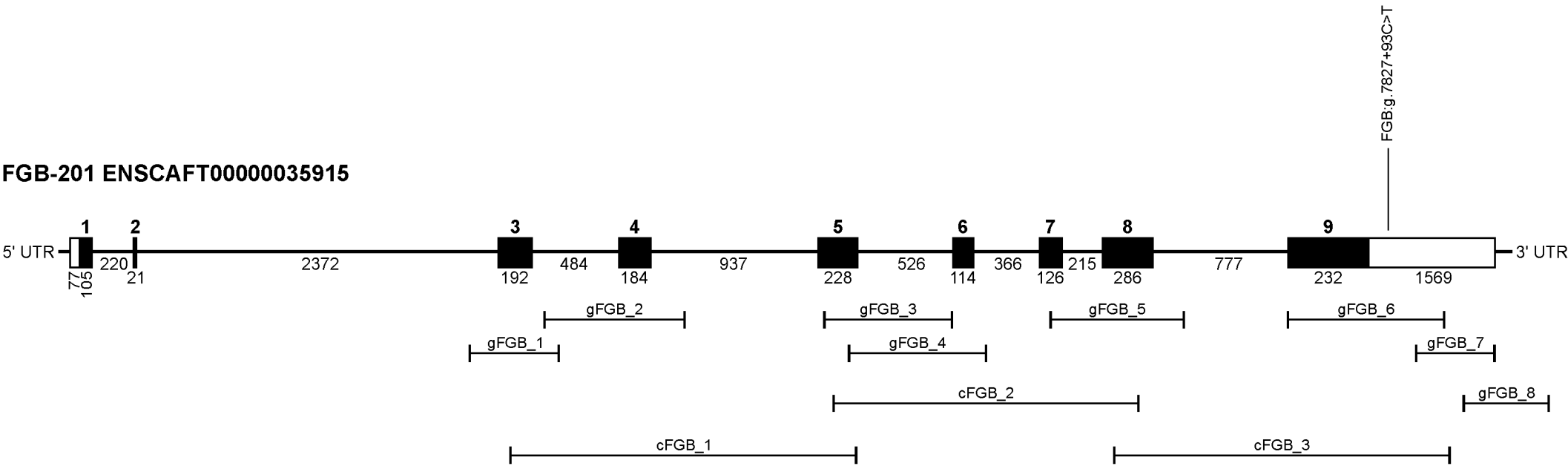

**FGB-202 ENSCAFT00000013386**

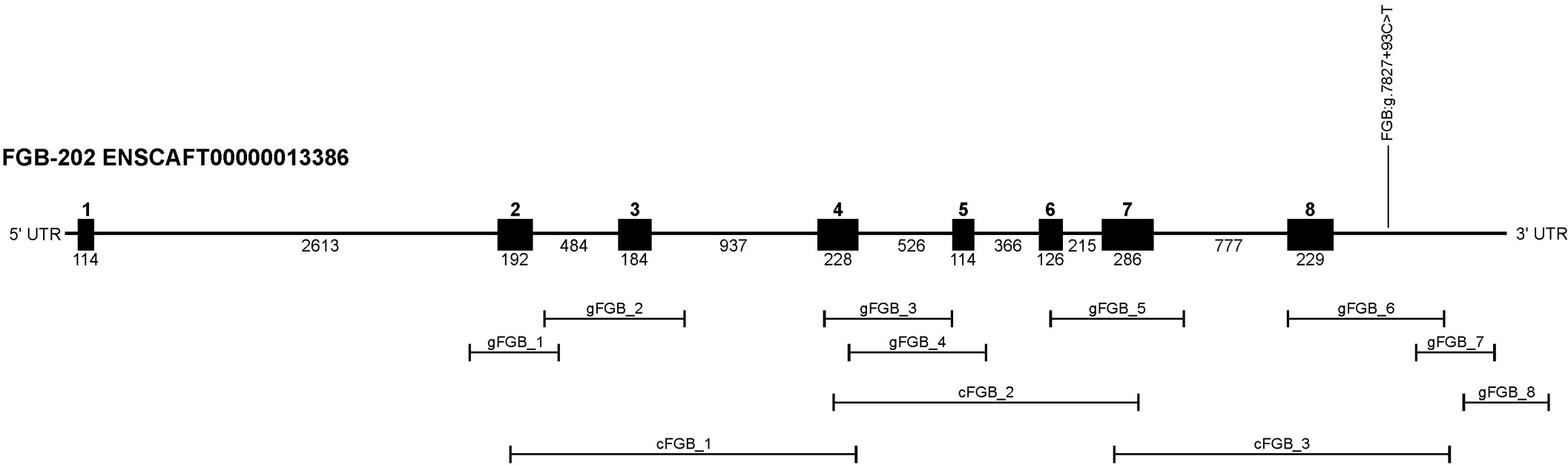

Supplement: Supplementary file 1 [file genes-12-01065-s001.zip › Figure_S1_FGB-Dog.pdf]

FGG-201 ENSCAFT00000013420

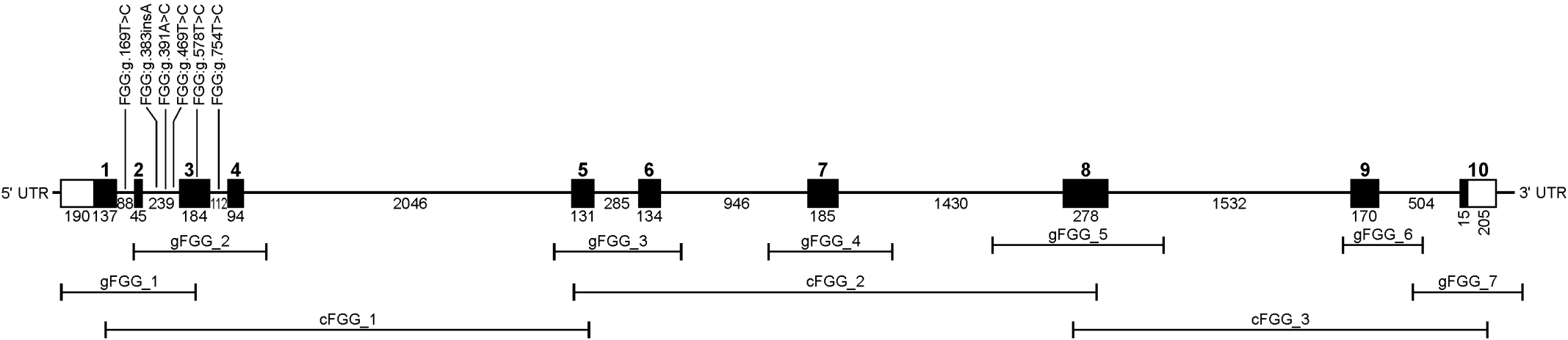

FGG-X2 XM\_532698.5

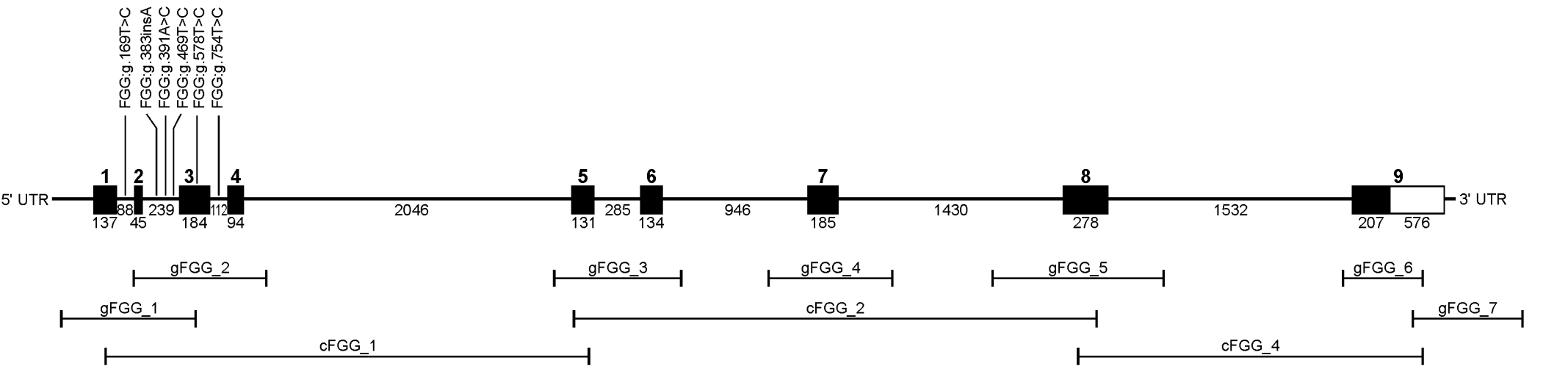

Supplement: Supplementary file 1 [file genes-12-01065-s001.zip › Figure_S2_FGG-Dog.pdf]
